# Subclinical recurrent neck pain and its treatment impacts motor training-induced plasticity of the cerebellum and motor cortex

**DOI:** 10.1371/journal.pone.0193413

**Published:** 2018-02-28

**Authors:** Julianne K. Baarbé, Paul Yielder, Heidi Haavik, Michael W. R. Holmes, Bernadette Ann Murphy

**Affiliations:** 1 Division of Neurology, Krembil Research Institute, University Health Network, Toronto, Ontario, Canada; 2 Institute of Medical Science, Faculty of Medicine, University of Toronto, Toronto, Ontario, Canada; 3 Faculty of Health Sciences, University of Ontario Institute of Technology, Oshawa, Ontario, Canada; 4 Faculty of Health, School of Medicine, Deakin University, Waurn Ponds, Victoria, Australia; 5 Centre for Chiropractic Research, New Zealand College of Chiropractic, Mount Wellington, Auckland, New Zealand; 6 Department of Kinesiology, Faculty of Applied Health Sciences, Brock University, St. Catharines, Ontario, Canada; Shanghai Mental Health Center, CHINA

## Abstract

The cerebellum processes pain inputs and is important for motor learning. Yet, how the cerebellum interacts with the motor cortex in individuals with recurrent pain is not clear. Functional connectivity between the cerebellum and motor cortex can be measured by a twin coil transcranial magnetic stimulation technique in which stimulation is applied to the cerebellum prior to stimulation over the motor cortex, which inhibits motor evoked potentials (MEPs) produced by motor cortex stimulation alone, called cerebellar inhibition (CBI). Healthy individuals without pain have been shown to demonstrate reduced CBI following motor acquisition. We hypothesized that CBI would not reduce to the same extent in those with mild-recurrent neck pain following the same motor acquisition task. We further hypothesized that a common treatment for neck pain (spinal manipulation) would restore reduced CBI following motor acquisition. Motor acquisition involved typing an eight-letter sequence of the letters Z,P,D,F with the right index finger. Twenty-seven neck pain participants received spinal manipulation (14 participants, 18–27 years) or sham control (13 participants, 19–24 years). Twelve healthy controls (20–27 years) also participated. Participants had CBI measured; they completed manipulation or sham control followed by motor acquisition; and then had CBI re-measured. Following motor acquisition, neck pain sham controls remained inhibited (58 ± 33% of test MEP) vs. healthy controls who disinhibited (98 ± 49% of test MEP, *P*<0.001), while the spinal manipulation group facilitated (146 ± 95% of test MEP, *P*<0.001). Greater inhibition in neck pain sham vs. healthy control groups suggests that neck pain may change cerebellar-motor cortex interaction. The change to facilitation suggests that spinal manipulation may reverse inhibitory effects of neck pain.

## Introduction

The neck is linked biomechanically and neurologically to the upper limbs, and yet, we know little about the mechanisms by which altered sensory feedback from the neck due to pain, fatigue, and altered posture affects upper limb sensorimotor integration (SMI) and the ability to learn new motor skills [1–4]. Motor learning refers to the acquisition or improvement of a motor skill with practice [5]. The cerebellum is known to undergo neuroplastic changes following motor training and is responsible for modulation of motor circuitry [6]. It plays a key role in processing sensory input to predict sensory consequences of movement for online motor corrections as well as for updating body schema in feedforward models of motor control [7], which allows corrections to be made prior to the time physically needed to receive sensory feedback from distal sources such as the hand [8].

Neck pain has been shown to influence sensorimotor function in older adults [9]. In the aforementioned study, the authors evaluated a range of sensorimotor functional tests and took into account other conditions that participants suffered from, yet found that the older adults with neck pain performed significantly worse than older adults who did not have neck pain [9]. The authors suggested that altered sensory modulation from the neck to the central nervous system (CNS) was likely responsible for the poor sensorimotor performance observed in this group [9]. Motor consequences of pain are considered to be integrated via thalamocortical-basal ganglia loops [10] and cerebellothalamocortical loops [11]. In the cerebellum, right and left lobules VI and VIIb demonstrate greater fMRI activation during pain and motor processing, in correlation with parallel activation of the thalamus and supplementary motor area [12]. Patients with cerebellar infarct showed greater sensitivity to experimental pain and poor motor coordination on cerebellar tasks [13]. The reciprocal role of the cerebellum to integrate sensory inputs and coordinate movements supports the proposition that painful inputs from the neck are likely to result in altered cerebellar integration and expression of motor outputs.

Transcranial magnetic stimulation (TMS) can be used to treat and measure the nervous system [14–17]. Functional connectivity between the cerebellum and motor areas can be assessed with TMS applied over the cerebellum followed 5–8 milliseconds later by TMS over the contralateral motor cortex (M1), which results in an inhibited motor response in distal hand muscles as compared to the motor response produced by TMS over M1 alone. This inhibited response is called cerebellar inhibition (CBI) [18,19]. Motor sequence training in healthy controls results in reduced CBI across a range of conditioning stimulus intensities [20].

The presence of neck pain alters both neck and limb sensorimotor function and motor control [9], and even milder forms of neck dysfunction can impact sensorimotor function [21,22]. These studies were performed with subclinical neck pain participants, or people with untreated mild-to-moderate recurrent neck pain [23,24]. Such recurrent pain represents a promising model to investigate long term consequences of altered sensory input from the neck on SMI. Whether subclinical recurrent neck pain alters motor learning is currently unknown. If this is the case, it could help explain why maladaptive motor patterns are maintained, potentially setting up a cycle of recurrent and chronic pain. There is a large body of evidence that reveals structural and functional changes within the CNS of people with chronic musculoskeletal disorders [25]. These changes may initially be beneficial, but as they persist they are thought to be influential in the pathophysiology of the condition and the developmental recurrence and maintenance of chronic symptoms [25].

Neuroplastic changes within different areas of the CNS are likely to help explain the transition from acute to recurrent to chronic conditions, sensory-motor findings, perceptual disturbances, why some individuals continue to experience pain when no structural cause can be discerned and why some fail to respond to conservative interventions in subjects with chronic musculoskeletal disorders [25].

One common treatment for neck pain is manual therapy, and in particular, spinal manipulation. Spinal manipulation alters firing from paraspinal muscles spindles [26], increases pain thresholds [27], changes functional connectivity in brain areas that process pain [28], and can alter motor output [22,29]. Spinal manipulation therefore, may affect SMI and cerebellar-cortical interactions. This study sought to explore whether subclinical recurrent neck pain influences cerebellar-M1 functional connectivity by comparing CBI responses before and after a novel motor sequence task in subclinical recurrent neck pain and healthy participants and by considering whether treatment with spinal manipulation changes the CBI response to motor acquisition. We hypothesized that healthy subjects would reduce CBI following motor acquisition (as previously found by Baarbé et al. [20], and subclinical recurrent neck pain subjects would have less reduced CBI following motor skill acquisition. We further hypothesized that spinal manipulation would restore CBI to levels similar to healthy controls.

## Methods

### Ethical approval

Each participant provided informed consent both in writing and orally, and the study was performed in keeping with the human right principles set out in the Declaration of Helsinki [30]. Ethics approval for the procedures were obtained from the University of Ontario Institute of Technology (UOIT) Research Ethics Board.

### Subjects

Twenty-seven subjects (16 females and 11 males; range, 18–27 years old; mean age ± SD, 21.1 ± 1.9 years) with recurrent mild neck pain and muscle tension, but minimal acute pain on the day of testing, participated in the study. Only untreated cases were studied, and as such these cases fit criteria previously described for subclinical neck pain [3,21,23,24,31–33]. The degree of pain and/or discomfort resulting from neck muscle tension was graded with the Chronic Pain Grade Scale [34,35]. This scale categorizes average pain intensity and disability over six months between Grade 0, meaning the participant experienced minimal neck pain and no disability in the previous six months, to Grade IV, meaning the participant experienced severe pain intensity and disability in the previous six months. Inclusion required a rating between Grade I to III, with pain- or discomfort-free days when they could be tested. People who were in extreme pain (e.g. Grade IV) were intentionally not tested so that pain during movement would not limit task performance. Rather, we sought to minimize the confounding effect of pain on movement patterns, as the intention of this study was to investigate the underlying changes resulting from ongoing changes in sensory information from the neck. Such patients would also have extreme difficulty sitting through several hours of the experiment.

Spinal manipulation or sham control were first randomized and then blindly allocated to the neck pain participants. Fourteen participants (8 females and 6 males; range, 18–27 years old; mean age ± SD, 21.2 ± 2.2 years) were part of the spinal manipulation group. Thirteen participants (8 females and 5 males; range, 19–24 years old; mean age ± SD, 21.0 ± 1.6 years) formed the sham control group. Participants were all right-handed, and had a mean ± SD score on the Edinburgh Handedness Inventory [36] of 73.9 ± 19.8 in the neck pain sham control group and 76.8 ± 20.7 in the spinal manipulation group.

Data were also collected from twelve healthy controls (2 females and 10 males; range, 20–27 years old; mean age ± SD, 22.4 years ± 2.2 years) who were pain free during testing and had no past history of neck pain or tension. Healthy participants were also right-handed, and had a mean ± SD score of 65.0 ± 31.8 on the Edinburgh Handedness Inventory.

### Neck pain characteristics

Frequency, duration, and location of neck pain, as well as the severity of neck pain were documented. To measure severity of neck pain, participants were shown a continuous 10 cm visual analog scale (VAS) and were asked to mark the severity of worse pain experienced in the previous six months. These scores were collected at the time of enrollment along with the Chronic Pain Grade Scale Grade [34,35].

### Exclusion criteria

Exclusion to participate included major structural injuries or anomalies to the cervical spine including disk herniation or fracture. As well, participants were excluded if they had received manual therapy or care for their neck in the previous three months. Exclusion for TMS included prior head injury, history of epilepsy, prior heart condition, recent intake of neuroactive medication, pregnancy, and metal implants in the head or upper body (e.g. pacemakers). Other exclusion items included inflammatory or system conditions (e.g. rheumatoid arthritis or infection), intake of anti-coagulant medication or bleeding disorders, vertigo or dizziness. Participants were additionally screened using a clinical practice guideline to manage the theoretical risk of vertebra-basilar stroke [37]. Participants were excluded who: 1) had a history of cervical artery dissection; stroke; acute neck, occipital or head pain that is severe and unlike any previously experienced; 2) had existing vertebral artery disease as evidenced by any one of the following: unilateral facial paresthesia, objective cerebellar defects, lateral medullary signs or symptoms (i.e. dysphagia, dysphonia, dysarthria, diplopia, ataxia, vertigo, nystagmus, hemianesthesia or unilaterally narrow pupil) or visual field defects; 3) Active cervical spine cord injury; 4) Acute cardiac disease.

### Experimental protocol

**Fig 1:**
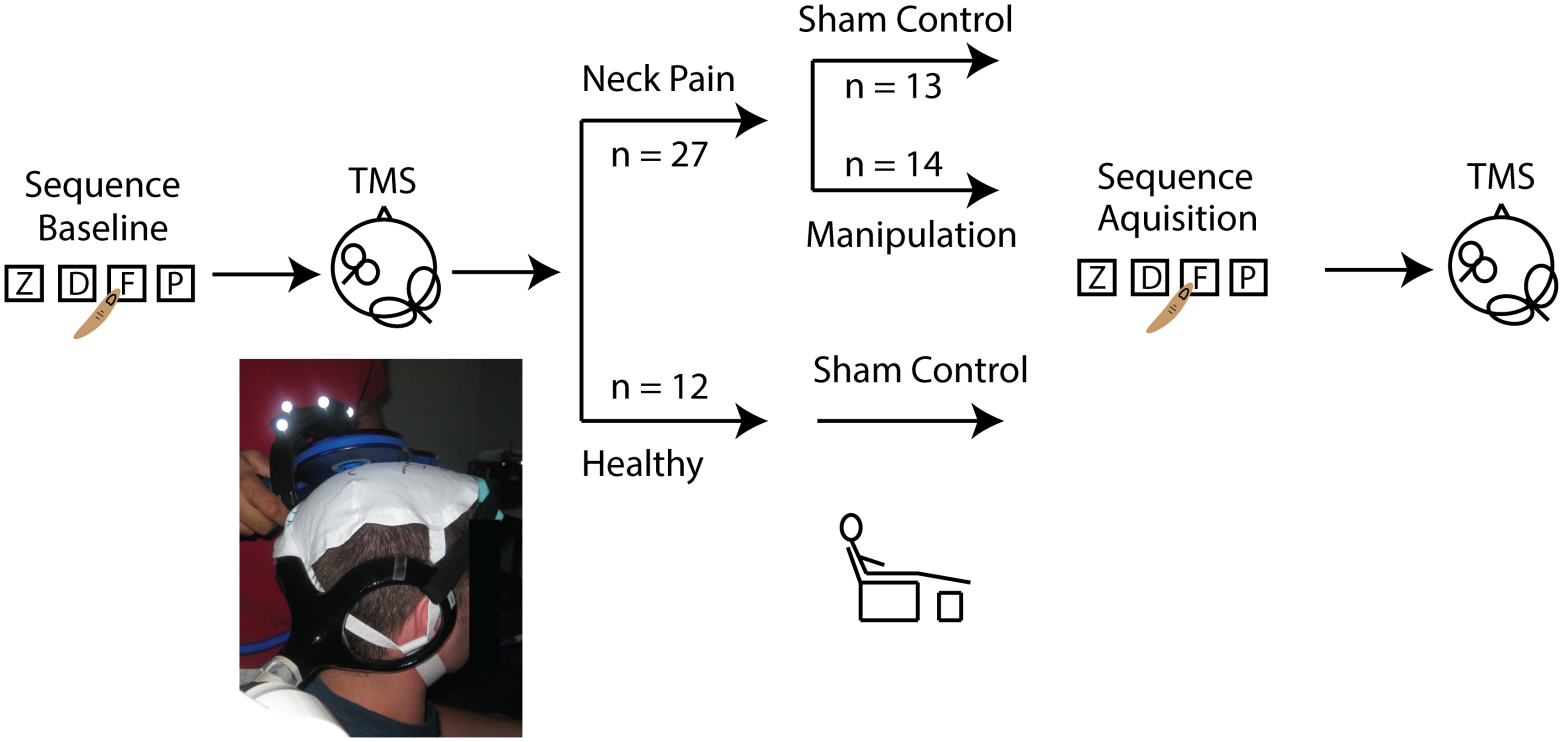
Experiment workflow in which CBI was measured before and after an intervention. The intervention consisted of spinal manipulation or sham control (~5–10 minutes) and motor sequence learning (~15 minutes).

All participants attended a session in which CBI was measured before and after the combined intervention (Fig 1). The CBI technique involved application of TMS over the cerebellum, ipsilateral to the right hand, and over M1 contralateral to the right hand. When TMS is applied over the left M1, a muscle twitch volley is produced and recorded from the right distal hand muscle. The combination of cerebellar and M1 stimulations at an inter-stimulus interval 5–8 milliseconds apart causes inhibition of the M1 muscle twitch volley [19,38].

Participants sat upright on a chair with their right arm resting on a pillow on their lap. Motor evoked potentials (MEPs) were recorded from the first dorsal interosseus of the right hand using disposable bipolar Ag/AgCl electrodes (Meditrace 130, Kendall, Mansfield, MA, USA). MEPs were amplified (x 1000) and band-pass filtered (20–1000 Hz) through a Cambridge Electronic Design 1902 amplifier (CED, Cambridge, England), sampled at 5 KHz (CED 1401, Cambridge, England) and recorded as a digital signal (Signal 4.08, CED, Cambridge, England).

Two TMS coils were used in the experiment. The first coil, a double cone coil (11 cm diameter), was held over the cerebellum, ipsilateral to the right hand, at the midline between the inion and the external auditory meatus at the level of, or slightly above the level of, the inion to elicit optimal MEP suppression as described by Ugawa et al. [19] (Fig 1). This coil connected to a Magstim Bistim (Magstim Co., Whitland, Dyfed, UK) which channels the voltage from two Magstim 200^2^ units together so that the output is 113% greater than output from a single unit [39]. The second coil was a figure-of-eight coil (9 cm diameter) held over M1 on the contralateral (left-hand) side and was held in a posterior direction approximately 45 degrees from the sagittal plane. This coil was connected to a Magstim 200^2^ (Magstim Co., Whitland, Dyfed, UK) and stimulator output was adjusted to elicit test MEPs ~ 0.5 millivolts (mV) in peak-to-peak amplitude. A test MEP of ~0.5 mV in peak-to-peak amplitude was selected since test MEPs less than 1 mV have been shown to be the most consistent for showing cerebellar inhibition [18,19].

### Cerebellar inhibition

Test MEP amplitude was found from the average of sixteen MEPs elicited by the figure-of-eight coil over M1. CBI was elicited by firing of the double-cone coil 5 ms in advance of the figure-of-eight coil. Ten cerebellar-M1 MEPs were elicited at 5% intensity increments of the cerebellar coil in ascending order (on average five cerebellar intensities were tested depending on when inhibition had reached 50 percent). Offline CBI_50_ was determined as the stimulator output of the double cone coil that would elicit a MEP ~50 percent of the test MEP alone, as described in Baarbé et al. [20]. CBI_50_ during the experiment was estimated, but to ensure that the stimulus for 50 percent level of CBI was not over- or under-estimated (a finding reported by Fisher et al. [40], additional CBI was measured at stimulator intensities above CBI_50_. Once CBI had been collected, resting motor threshold (RMT) was determined as the stimulator intensity that would elicit five out of ten MEPs with peak-to-peak amplitudes equal to or greater than 50 microvolts (μV). This entire protocol, with the inclusion of RMT and test MEP collections, was repeated before and after the intervention of manipulation or sham control and upper limb motor acquisition.

### Spinal manipulation

A practicing registered chiropractor applied spinal manipulation to the regions of palpable tenderness and restricted movement in the cervical spine [41–44]. The manipulations performed involved high-velocity, low-amplitude spinal manipulation. All treated areas of the neck region were documented and recorded on the participant’s file alongside the frequency, location, duration and severity described by the participant. For the duration of the brief time of the intervention or control (five to ten minutes), the participant was moved to a reclining chair. Electrodes were kept on the participant, and the only change to the experimental setup was that the leads were unplugged from their acquisition units. Care was taken to ensure that the participant returned to the same posture in the experimental chair as before the intervention.

### Sham procedure

For the sham procedure, participants were moved to the reclining chair. Light palpation was applied to the neck, and the head was gently moved into lateral flexion and rotation in a similar manner to the actual neck manipulation, without applying the high-velocity, low-amplitude manipulative thrust. This condition was carried out to ensure that the changes that were measured after the manipulation were not simply due to the passage of time, or due to the activation of muscle spindles, joint receptors or cutaneous touch that would occur as the neck is positioned for cervical spine manipulation. All of the neck pain controls (*n* = 13) and eight of the 12 healthy controls received the sham procedure. All participants, regardless of their group, completed the motor sequence acquisition task before completing the final CBI measurements.

### Motor acquisition task

The motor acquisition task involved typing randomized eight-letter sequences of the letters Z, P, D, F (i.e. Z, D, P, Z, F, P, D, D) as quickly and as accurately as possible (E-Prime 2.0, Psychology Software Tools, Sharpsburg, Pennsylvania). This task was performed on a specially-designed keyboard to encourage right index finger adduction-abduction to reach individual keys. The task took participants ~15 minutes to complete and was selected because similar tasks have been shown to activate the cerebellum [45,46]. Performance on the task was measured as the response time to press each key sequentially as well as the accuracy of each key selection. To evaluate performance, participants completed a short test block (~1–2 minutes) comprised of ten instances of eight-letter sequences. Participants completed this short test block at the beginning of the study before the TMS collection. Following TMS and spinal manipulation/sham control, participants then completed another short test block followed by a longer motor acquisition block (~10 minutes) and a final short test block. Immediately upon completing the motor acquisition task, we re-measured CBI. The entire length of the motor acquisition task (~15 minutes) was a duration that did not fatigue the participants, yet produced significant practice effects [20]. A brief time elapsed (~5 minutes) from the time that participants finished typing to when CBI was retested, during which time we repositioned the double-coned coil over the cerebellum and rested MEPs over M1 alone. Electrodes placed on the first dorsal interosseous recorded background muscle activity while completing the task.

### Data analysis

As described previously [20,47], the traces were carefully examined at high gain for instances of cervicomedullary evoked potentials (CMEPs), cervical root activity, and antidromic activity, which were considered extraneous to the experiment objectives. Previous work had identified CMEPs elicited from double-cone stimulations over the inion and recorded from the first dorsal interosseous to have a latency of ~18 ms and cervical root activity to have a latency of ~15 ms [47]. Whereas, CBI ideally should elicit inhibited MEPs that have a latency ~21 ms from stimulation of M1 [47] which in our experiments was ~26 ms from stimulation over the cerebellum, considering the 5 ms interval from the cerebellum stimulus (double-cone coil) to the M1 stimulus (figure-of-eight coil). Individual traces that showed extraneous activity, along with traces that showed background muscle activity before or following stimulation, were removed and the remaining MEPs (in most cases 10 MEPs, but no fewer than 7) were averaged for each level of stimulus intensity.

Average peak-to-peak amplitude was found for each level of stimulus intensity and divided by the test MEP for each participant for each intensity of CBI. CBI at 50 percent (CBI_50_) was used as the level for comparisons as described in Baarbé et al. [20]. CBI_50_ represents the stimulator intensity that elicits MEPs that are ~50 percent inhibited. Conditioning stimulus intensities 5% and 10% above CBI_50_ were also evaluated (CBI_50_+5% and CBI_50_+10%), as these intensities showed reduced CBI following motor sequence acquisition in healthy controls [20].

To evaluate motor-training induced plasticity, response time and accuracy was assessed across groups before and after the motor acquisition task. Accuracy to perform the motor sequence task was calculated out of a maximum accuracy response of 80 (score of 72 = 90% accuracy). Values are reported as mean ± 1 SD in the text and mean ± 1 SEM in figures.

### Statistics

The frequency, severity, duration of neck pain and Chronic Pain Grade Scale between sham and treatment recurrent neck pain groups were evaluated with independent-samples *t* tests. To assess changes to test MEP amplitude and RMT pre- and post-intervention, a 3 x 2 mixed-design repeated-measures ANOVA was applied. The between-subjects factor was group (neck pain sham control, neck pain manipulation, and healthy controls) and the repeated within-subject variable included time (pre- and post-intervention).

We then applied a repeated-measure, 3 x 2 x 3 mixed-design analysis of variance (ANOVA) to assess CBI change across the three groups. The between-subjects factor was group (neck pain sham control, neck pain manipulation, and healthy controls) and the repeated within-subject variables included time (pre- and post-intervention) and stimulator intensities (CBI_50_, CBI_50_+5% and CBI_50_+10%). Post-hoc Welch’s one-way ANOVA and Welch’s *t* tests were used to assess post-intervention CBI differences between the three groups [48]. Comparisons before and after the intervention were assessed with paired *t* tests, and Bonferroni correction was applied for multiple comparisons. Relationships between mean CBI change and pain severity were assessed with Spearman rank-order correlation.

Mean response time on motor sequence acquisition was assessed with a repeated-measures, 3 x 3 mixed-design ANOVA. The between group factor included group (neck pain controls, neck pain manipulation, and healthy controls), and the repeated within group factor included time (baseline at the start of the experiment, pre-task condition, and post-task condition). Accurate and inaccurate responses for each group were tallied using a 6 column, 2 row χ^2^ test to evaluate accuracy immediately before and after the task. The alpha value was set to 0.05 for all statistical tests, and statistical analysis was performed using SPSS (V22, International Business Machines Corporation, Armonk, New York).

## Results

### Neck pain characteristics

The neck pain characteristics for the manipulation and neck pain control groups are summarized in Table 1. Independent-samples *t* tests showed the two groups did not differ in measures for frequency of pain episodes, severity of pain, duration of recurrent neck pain, and Chronic Pain Grade Scale scores. The number of segments manipulated for individual participants ranged from 2 to 4 spinal segments between C1 and T1 vertebrae on either side of the neck (depending on which side met the criteria for manipulation described above).

**Table 1:** Neck pain characteristics reported as mean ± standard deviation for each group.

	*Pain Controls*	*Manipulation*	*Healthy*	*Pain vs. Healthy*†	*Pain Control vs. Manipulation*†
Frequency of neck pain (days per month)	16.9 ± 9.1	14.5 ± 8.8	1.0 ± 1.0	<0.001	0.5
Duration of neck pain (years)	1.9 ± 1.5	3.2 ± 2.2	**	**	0.09
Most severe pain intensity in previous 6 mo (10 cm VAS)	5.6 ± 2.1	6.4 ± 2.1	1.4 ± 1.6	<0.001	0.4
Chronic Pain Grade Scale	1.5 ± 0.7	1.7 ± 0.6	**	**	0.3
Disability Days (average in previous 6 mo)	2.2 ± 3.3	5.4 ± 8.2	**	**	0.2

^†^values reported as *P* values from independent sample *t* tests

** not applicable

VAS, visual analog scale presented as a continuous line

### Cerebellar inhibition

The mean test MEP amplitude before the intervention was 0.67 ± 0.12 mV and 0.64 ± 0.16 mV following the intervention. Test MEP amplitudes showed no interaction effects of group vs. time (*F*_2,36_ = 1.332, *P* = 0.3) or effects of time (*F*_1,36_ = 1.241, *P* = 0.3). RMT data was missing from one healthy control and two neck pain sham controls. However, in the remaining subjects (14 neck pain manipulation, 12 neck pain sham controls, and 11 healthy), RMT showed no interaction effects of group vs. time (*F*_2,33_ = 0.297, *P* = 0.7) or effects of time (*F*_1,33_ = 0.274, *P* = 0.6). The mean RMT before the intervention was 43.3 ± 7.5% of maximal stimulator output and 43.0 ± 7.0% maximal stimulator output following the intervention. Mean test MEP stimulator intensity was 48.6 ± 8.4% maximal stimulator output before the intervention and 48.8 ± 8.3% after the intervention.

**Fig 2:**
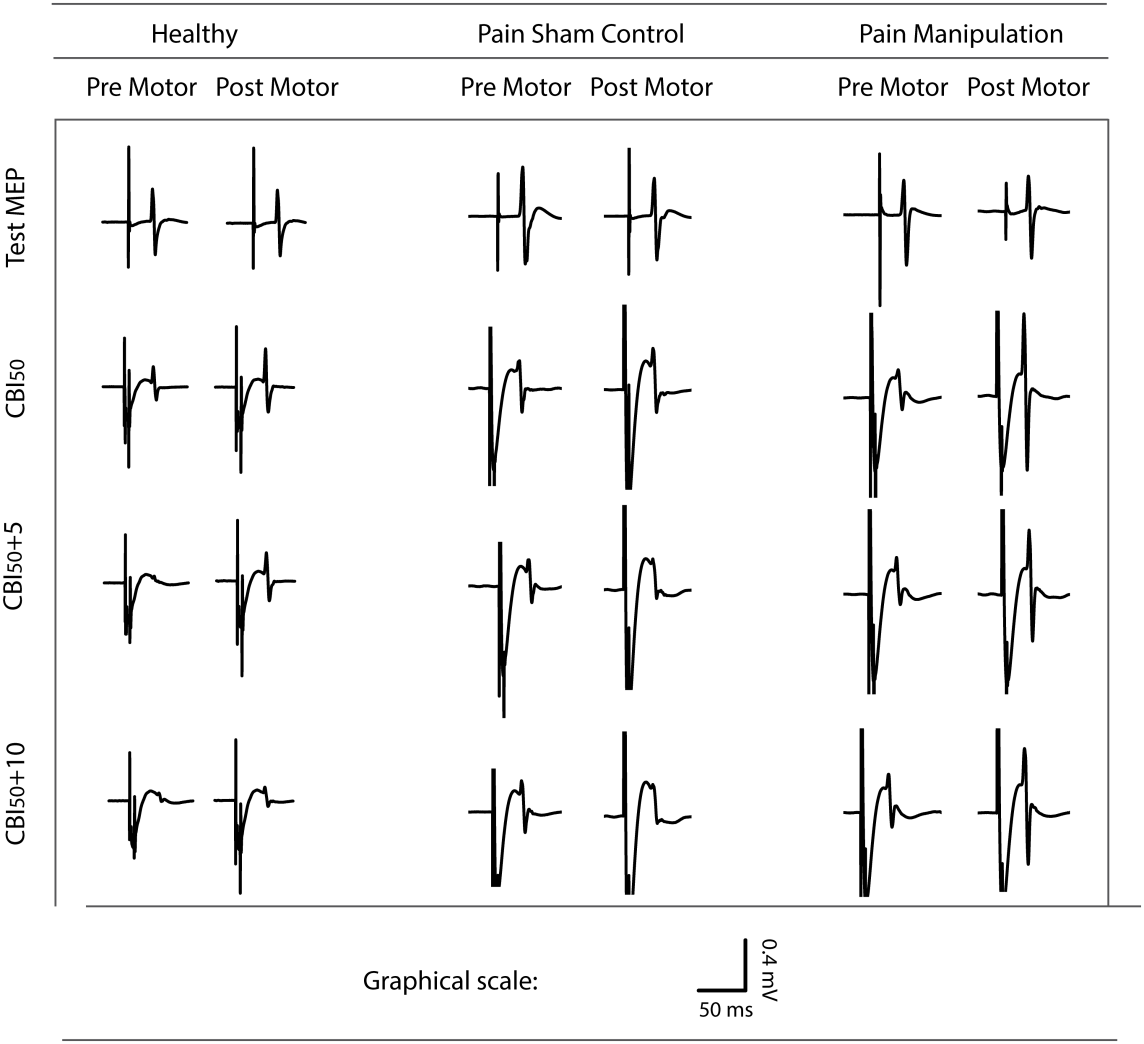
Raw traces from representative participants from all three groups. Here, CBI_50_ is depicted pre- and post-spinal manipulation or sham control and motor training, as relative to raw traces of M1 activation. Pre motor, prior to spinal manipulation/sham control and motor sequence acquisition; Post motor, following spinal manipulation/sham control and motor sequence acquisition.

**Fig 3:**
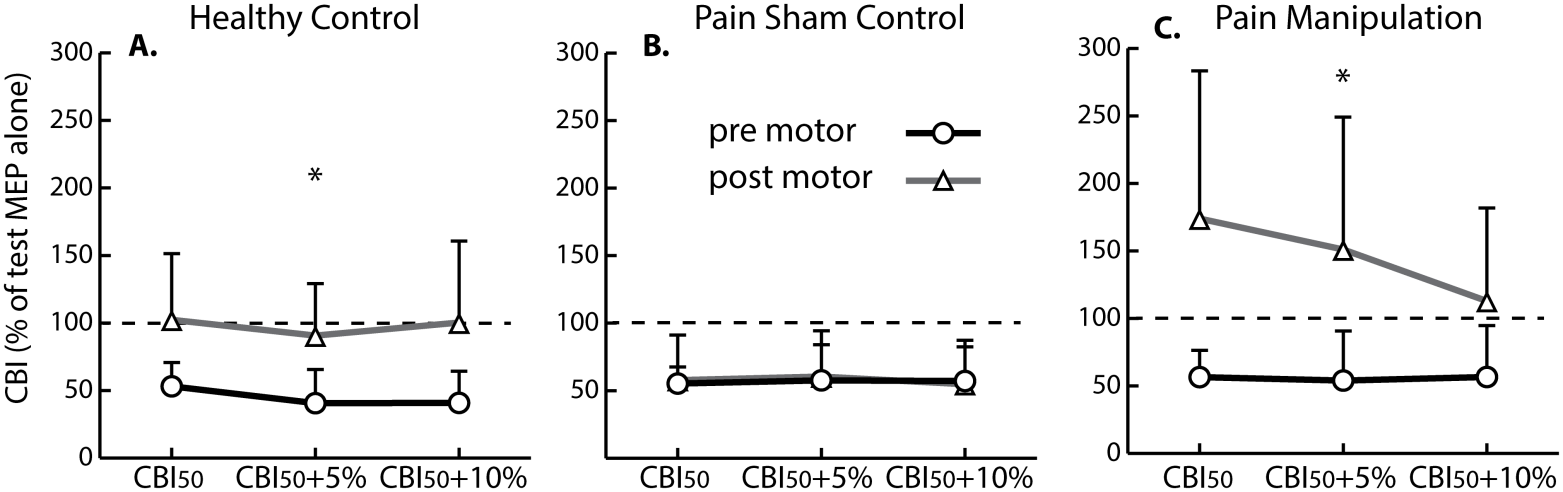
Mean CBI_50_ responses in each group. (A) Mean CBI_50_ response in healthy controls before and after the combined intervention of sham control and motor sequence acquisition. (B) Mean CBI_50_ response in neck pain control group before and after the combined intervention of sham control and motor sequence learning. (C) Mean CBI response in spinal manipulation group before and after the combined intervention of spinal manipulation and motor sequence learning. Pre motor, prior to spinal manipulation/sham control and motor sequence acquisition; Post motor, following spinal manipulation/sham control and motor sequence acquisition. Error bars depict SEM. *P < 0.001.

CBI traces from representative subjects from all three groups can be found in Fig 2. In the pre-intervention state, participants showed a CBI response ~50 percent of the original test MEP. Following the intervention, the neck pain sham control showed no change to CBI_50_ (remained inhibited), the healthy participant had reduced CBI_50_, and the neck pain spinal manipulation subject had even greater reduction to CBI_50_. The same pattern emerged in the group data such that neck pain sham controls remained inhibited following motor acquisition whereas healthy controls and neck pain spinal manipulation dis-inhibited (Fig 3 and S1 Table).

A significant main effect of time (*F*_1,36_ = 37.378, *P* < 0.001, Cohen’s *d* = 2.04) emerged as well as a significant interaction of group vs. time (*F*_2,36_ = 11.247, *P* < 0.001, Cohen’s *d* = 1.58). *Post hoc* Welch’s *t* tests showed significantly less CBI following motor acquisition in the healthy group compared with the neck pain sham control group (*P* < 0.001; healthy controls, 98 ± 49% of test MEP; neck pain sham controls, 58 ± 33% of test MEP). The manipulation group also showed significantly less CBI compared with the neck pain control group (*P* < 0.001; manipulation, 146 ± 95% of test MEP; neck pain sham control, 58 ± 33% of test MEP). When the spinal manipulation and healthy participants CBI levels were compared, the spinal manipulation group showed significantly less CBI than the healthy controls (*P* = 0.006; manipulation, 146 ± 95% of test MEP; healthy, 98 ± 49% of test MEP).

*Post hoc* paired *t* tests comparing pre- vs. post-intervention in each of the groups showed significantly less CBI following motor acquisition in healthy participants (an increase in magnitude from 45 ± 22% of test MEP pre to 98 ± 49% of test MEP post, *P* < 0.001). As well, the manipulation group showed significantly less CBI (an increase in magnitude from 56 ± 32% of test MEP pre to 146 ± 95% of test MEP post, *P* < 0.001). Neck pain controls, however, showed no changes to inhibition (*P* = 0.86; pre, 57 ± 21% of test MEP; post, 58 ± 33% of test MEP) (Fig 3).

**Fig 4:**
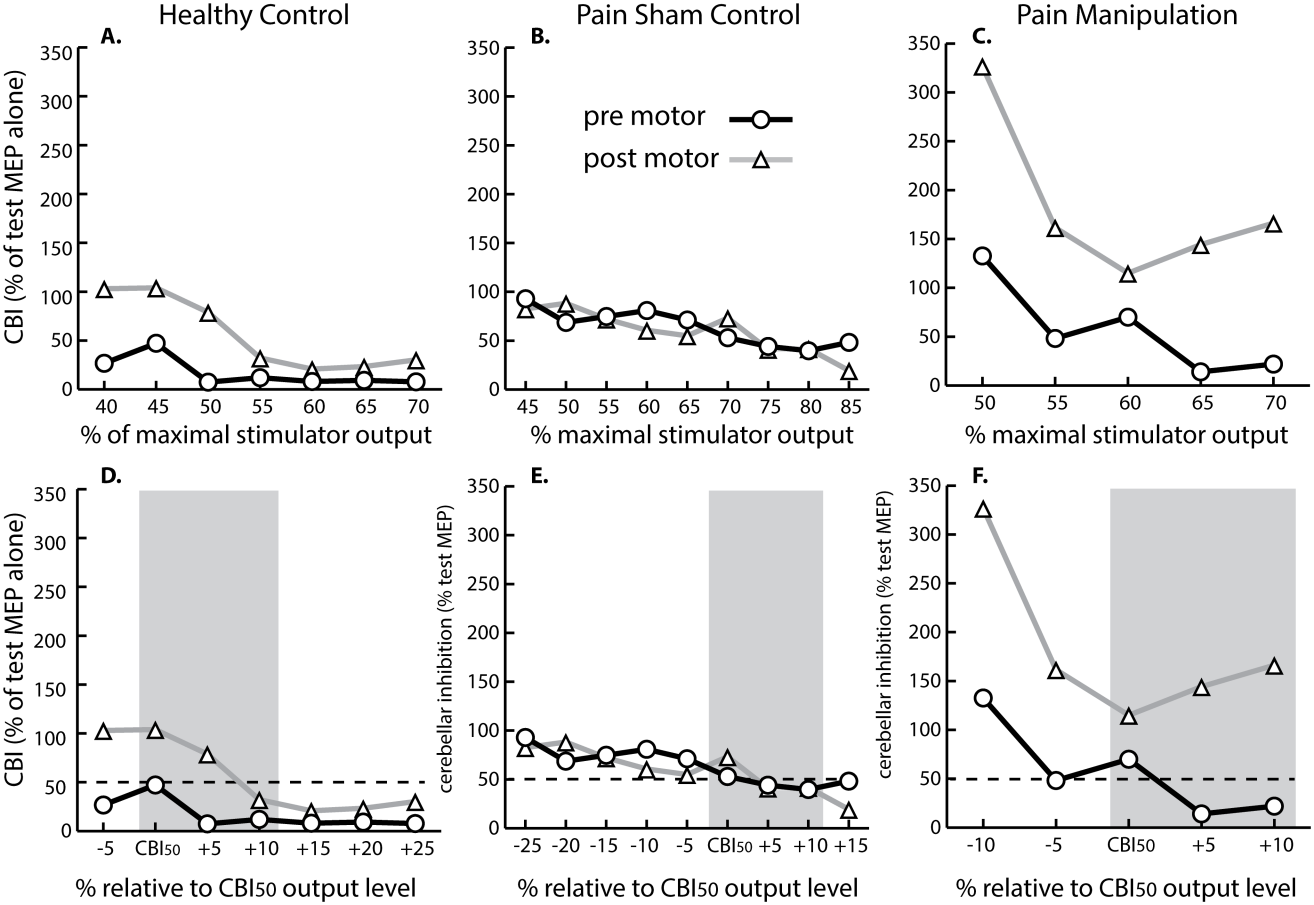
Stimulus-response curves for representative subjects. (A-C) Stimulus-response curves relative to maximum stimulator output on the x-axis for representative subjects that were (A) healthy, (B) recurrent neck pain sham, and (C) recurrent neck pain manipulation. (D-F) Stimulus-response curves relative to CBI_50_ as shown on the x-axis for representative subjects that were (D) healthy, (E) recurrent neck pain sham, and (F) recurrent neck pain manipulation. Pre motor, prior to spinal manipulation/sham control and motor sequence acquisition; Post motor, following spinal manipulation/sham control and motor sequence acquisition.

CBI measured at different stimulator intensities from representative subjects for each group are displayed in Fig 4. When stimulator intensities other than CBI_50_ were considered, the trend still emerged of less CBI in the healthy control and spinal manipulation groups and persistence of CBI in the neck pain sham control group following motor acquisition. Inter-subject variability of cerebellar responses can also be seen in Fig 4. In one participant, CBI_50_ was observed at intensities as low as 45% maximal stimulator output (Fig 4A), and in another participant, a stimulator intensity as high as 70% maximal stimulator output was necessary before CBI_50_ could be elicited (Fig 4B).

### Pain severity and CBI relationships

**Fig 5:**
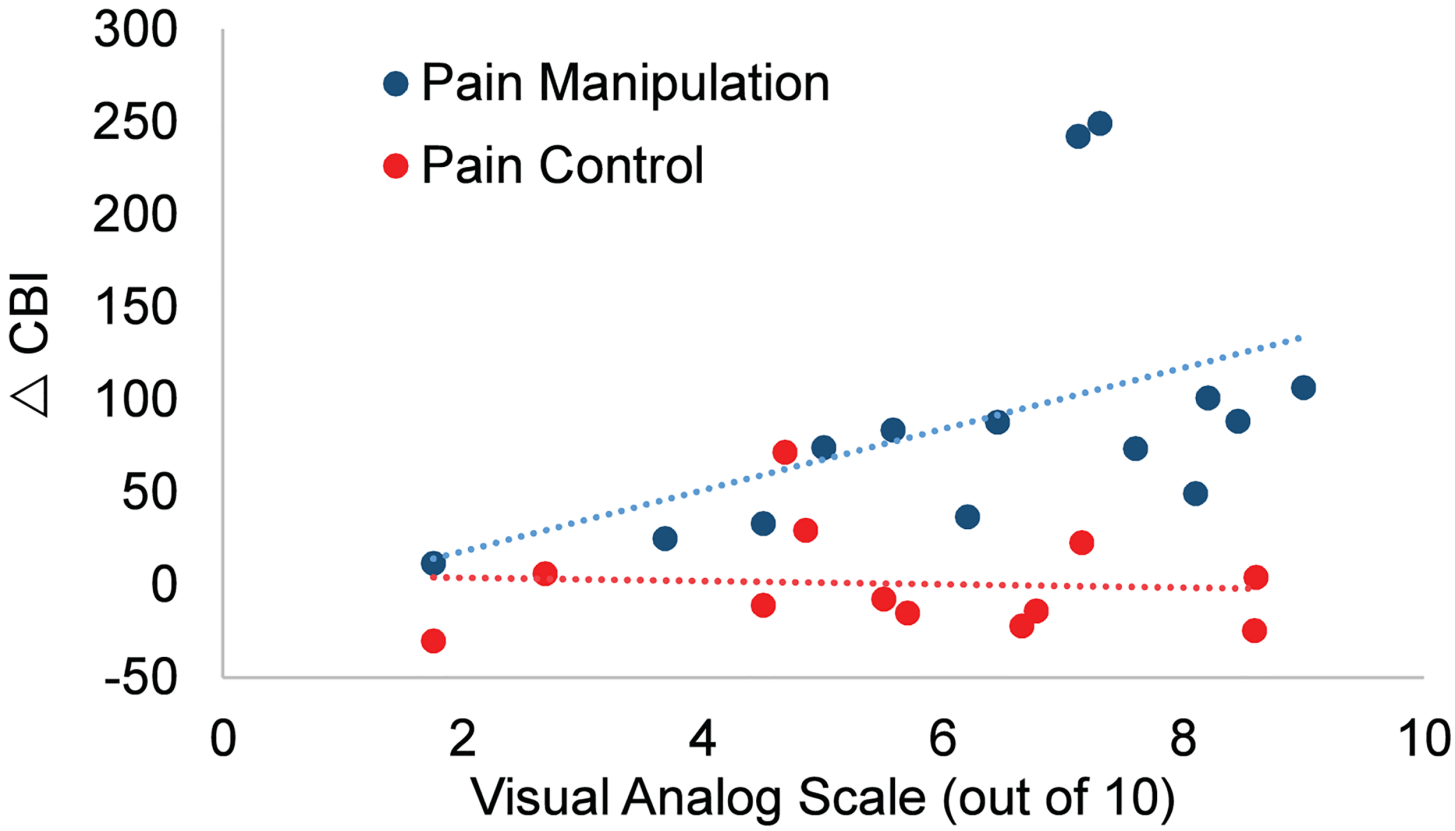
Relationship between CBI change and pain severity. Participants that had greater pain disinhibited CBI more following manipulation (blue) (*r*_*s*_(12) = 0.7, *P* < 0.01), but not following sham control (red).

A significant relationship was found between average overall pain severity during painful episodes in the previous six months and change to CBI in the manipulation group (*r*_*s*_(12) = 0.7, *P* < 0.01). The relationship was such that when neck pain was more severe, spinal manipulation subjects had greater CBI change. Neck pain sham controls, however, did not show this relationship (Fig 5).

### Motor acquisition

**Fig 6:**
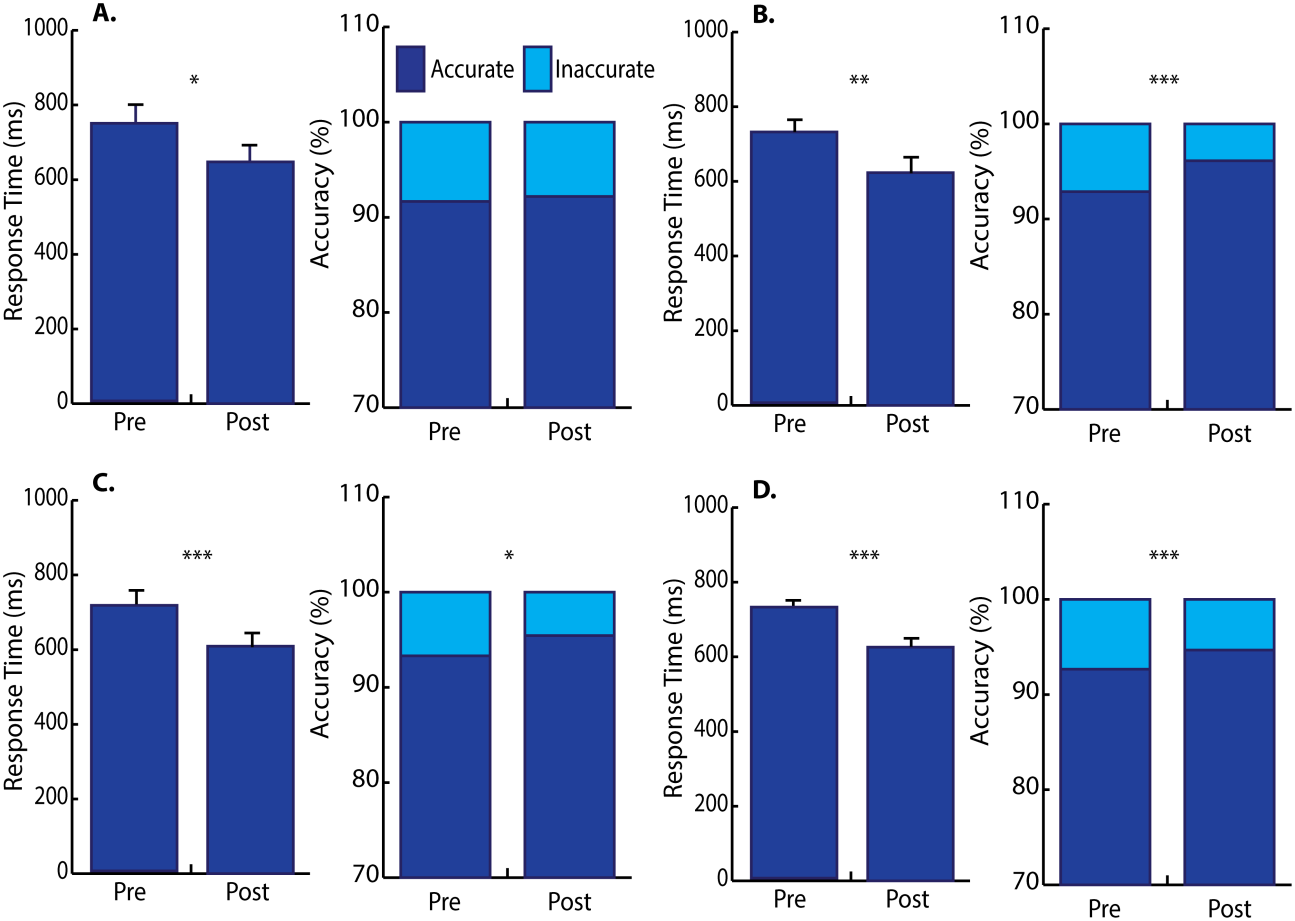
Mean response time and accuracy pre- and post-motor acquisition. (A) Healthy participants had marginally reduced response time and had no change to accuracy. (B) Neck pain sham control had moderate reduction to response time and dramatically improved accuracy. (C) Neck pain treatment dramatically reduced response time and had only marginal increase to accuracy. (D) All subjects demonstrated overall reduction to response time and accuracy from pre- to post-motor acquisition. Error bars depict 1 SEM. Pre, pre-motor acquisition; Post, post-acquisition. * *P* = 0.02–0.04; ** *P* = 0.005; *** *P* ≤ 0.001.

Fig 6 and S2 Table depict motor performance pre- and post-motor acquisition. Baseline motor performance was not collected in one healthy participant and that subject was excluded from comparisons involving baseline, but included in subsequent comparisons pre- and post-motor acquisition. A main effect of response time emerged across the three conditions (baseline, pre-task vs. post-task conditions) in all groups (healthy and 2 recurrent neck pain groups) (*F*_2,70_ = 51.563, *P* < 0.001, Cohen’s *d* = 2.43, no interaction effect). Post-hoc repeated tests were run on each group and the Bonferroni-corrected α level was set to 0.005 (10 comparisons). As Bonferroni correction reflects a conservative approach, unadjusted *P* values are reported here, both in the text and figure legend (Fig 6). Healthy participants showed a significant effect of time (*F*_2,20_ = 14.031, *P* < 0.001, Cohen’s *d* = 2.37). *Post hoc* paired *t* tests with Bonferroni correction showed marginal reduction to response time from baseline to the pre-task condition (869.4 ± 288.1 ms vs. 704.2 ± 151.6 ms, *P* = 0.009) and no change to response time from pre- to post-task conditions (743.6 ± 198.9 ms vs. 647.6 ± 153.3 ms, *P* = 0.034).

The neck pain control also showed a significant effect of time (*F*_2,24_ = 11.186, *P* < 0.001, Cohen’s *d* = 1.93). However, paired *t* tests showed no increases in speed from baseline to pre-task conditions which was the period of time when they received sham control (772.3 ± 178.8 ms baseline vs. 725.0 ± 143.8 ms pre-test, *P* = 0.20). Once motor training had been completed, neck pain controls showed increases in speed from the pre-task condition to the post-task condition (725.0 ± 143.8 ms pre-task condition vs. 623.3 ± 152.0 ms post-task condition, *P* = 0.005).

Manipulation participants showed a significant effect of time (*F*_2,26_ = 40.338, *P* < 0.001, Cohen’s *d* = 3.52), and paired *t* tests with Bonferroni correction showed their response times were significantly faster from baseline to pre-task conditions which was the period of time in which they received a manipulation. The increase in speed following the manipulation was from 800.2 ± 214.0 ms baseline to 711.3 ± 171.8 ms pre-task (*P* = 0.0026). Following completion of the motor training task, a dramatic increase in speed occurred from 711.3 ± 171.8 ms pre-task to 609.2 ± 158.0 ms post-task (*P* < 0.001).

Accuracy tests considered the times immediately before and after motor acquisition and showed a shift in accuracy following motor acquisition in all groups [χ^2^(5) = 28.173, *P* < 0.001]. *Post hoc* tests compared each group and the Bonferroni-corrected α level was set to 0.01 (5 comparisons). Healthy controls showed no significant improvement to accuracy at pre-motor acquisition vs. post-motor acquisition [χ^2^(1) = 0.175, *P* = 0.7]. Sham controls showed dramatic improvement to accuracy pre- vs. post-motor acquisition [χ^2^(1) = 10.728, *P* = 0.001]. In contrast, spinal manipulation participants did not show a significant change to accuracy, although they showed a trend to increase accuracy pre-motor training vs. post-motor training [χ^2^(1) = 4.844, *P* = 0.028] (Fig 6). No correlation was found between CBI and response time or accuracy in any group.

## Discussion

The novel finding in this study is that altered sensory input from the neck due to recurrent neck pain and its treatment affects the way in which the cerebellum interacts with M1 in response to motor skill acquisition. Subclinical recurrent neck pain subjects who received spinal manipulation had a 90% average reduction to CBI pre- vs. post-motor acquisition vs. a 50% average CBI reduction for the healthy group, and only a 1% average CBI reduction in the subclinical recurrent neck pain group who receive sham control. It is well known that the cerebellum receives and integrates large amounts of sensory information from joints, tendons and muscles, including those from the intervertebral regions of the neck [49] as well as nociceptive inputs [11]. These inputs to the cerebellum become integrated for timing of limb movements, and creation of forward internal models to predict the sensory consequences of movement milliseconds prior to the movement [8]. However, it has not previously been shown that improving neck function, such as with spinal manipulation, may actually alter cerebellum-M1 communication, or impact motor skill acquisition. Again, the cerebellum is well known to be highly active in motor learning and motor adaption [45,50,51]. Multimodal processing occurs in lobules VI and VIIb of the cerebellum, and these areas have been found to be important for pain-related adaptations in motor control [12]. The cerebellar response differences in the recurrent neck pain group suggest that their neck dysfunction alters the way their cerebellums function during motor acquisition tasks, and that this can be dramatically impacted by as little as a single spinal manipulation session.

The increased magnitude of the CBI response in the manipulation group (larger than M1 response alone) indicates that there may be a cumulative effect of other processes in the cerebellum or M1. This effect may be described as cerebellar facilitation (CBF, as opposed to reduced CBI). The presence of cerebellar stimuli 5 ms prior to M1 test stimuli has been shown to reduce short interval intracortical inhibition (SICI) and increase intracortical facilitation (ICF) [18], suggesting that the cerebellum has a unique interaction effect with inhibitory and excitatory neurons in the motor cortex. Similarly, cervical manipulation has unique effects on various cortical inhibitory and facilitatory mechanisms including decreased SICI, increased short-interval intracortical facilitation (SICF), and a lengthening of the cortical silent period (CSP) [22,52]. It is possible that the findings of CBF following spinal manipulation is due to interaction with these cortical changes. Noteworthy to consider is that these cortical changes were found without change to *F* wave persistence or amplitude following cervical spine manipulation [22], indicating that cortical or subcortical mechanisms mediate these effects rather than changes to spinal excitability. Another study has also shown that the motor output effects of spinal manipulation are likely attributed to increased descending drive [29]. The study found significant increases in maximum voluntary contraction forces (16% increase) following spinal manipulation, with matching large (45% increase) significant changes in volitional waves (*V* waves) yet only minimal (8.5% decrease) changes in the Hoffmann reflex (*H* reflex) threshold [29]. *V* waves depend upon the density of action potentials sent down from the supraspinal centers that collide with antidromic action potentials in motor axons caused by supramaximal stimulation of the innervating nerve. Thus, this study’s findings support the notion that spinal manipulation has a supraspinal mechanism likely involving cortical and/or cerebellar circuits.

Although M1 circuits (e.g. SICI, ICF, SICF, CSP) may be important for the observed findings, it is unlikely that direct changes to M1 excitability had a major contribution to the findings. MEP amplitudes and RMT did not change in any group pre- to post-intervention. The stimulator output for the test MEP was also the same pre- and post-motor training. If M1 excitability contributed to the CBI findings, we would expect to observe a systematic change to these measures of M1 excitability, which we did not see.

CBI was not reduced in neck pain controls. We propose that this represents a different recruitment of circuits in the cerebellum and/or motor cortex. TMS studies have found that chronic pain results in increased SICI (less amplitude) and decreased ICF (also less amplitude), even in the absence of structural pathology [53,54]. EEG showed that prefrontal gamma activity is modulated during pain, which may demonstrate a basic mechanistic deficit of inhibitory GABAergic neurotransmitters [55]. Pain is also found alongside changes to inhibition of muscles in the area of the pain including non-painful antagonistic and synergistic muscles [56]. However, the mechanism of these inhibitory changes is yet unclear. The basal ganglia may play a modulatory role on inhibitory output [10]. However, changes to basal ganglia function does not directly explain heightened activity of the cerebellum in the presence of acute and chronic pain states [11].

Acute experimental pain has also been shown to change indices of motor plasticity [57,58] without dramatically affecting task performance [56]. Unlike acute pain models, subclinical recurrent neck pain represents a chronic model of intermittent, yet ongoing, pain. The recurrent nature of the pain means that the impact of altered sensory input from the neck on cerebellar processing can be investigated without severe confounding effects of sudden acute pain [59,60]. Subclinical recurrent neck pain has been associated with decreased cervical kinesthesia, neck range of motion and neck muscle endurance [23,24,32]. Growing evidence suggests that neck pain in general has implications to sensorimotor function including losses to shoulder and neck proprioception [3,24,61] and poor smoothness of movement [62]. Thus, subclinical recurrent neck pain presents as a useful model of pain to study cerebellar changes and SMI implications.

The primary difference between the two neck pain groups was the type of intervention received prior to motor training. The manipulation group received spinal manipulation, whereas the neck pain controls had their necks passively moved to challenge the neck joints close to the end range and to mimic spinal manipulation, but without delivering the actual high-velocity, low-amplitude manipulative thrust. The control intervention was very similar to low level mobilizations delivered by many manual therapists. Indeed, participants reported feeling better after the control intervention suggesting that it was a good control for both therapist-patient interaction effects as well as the physiological effects of moving the neck passively. This suggests two things: 1) that this type of control intervention is well received by study participants and 2) that high-velocity, low-amplitude manipulation has a different physiological effect on the central nervous system compared to low level mobilizations.

We also found that all participants showed a similar significant practice effect on the motor acquisition (e.g. main effect of time) and no interaction effect between group and time, which indicates that the practice effect of each group did not significantly differ from each other. It is significant to note that the practice effects themselves did not differ significantly between groups, yet CBI following motor acquisition did differ among the three groups. Spinal manipulation seems to activate different circuits following motor acquisition as compared to the sham control intervention for the recurrent neck pain group.

All objective “physical” traits in the two recurrent neck pain groups including the frequency, duration and intensity of their ongoing recurrent neck pain showed no differences. The manipulation group had a significant relationship between pain severity and CBI change, whereas sham control subjects did not show such a relationship. The relationship between pain severity and CBI change in the manipulation group indicates that those with the most severe pain had the most activation of cerebellar and/or cortical circuits. The lack of a relationship in sham controls suggests that different circuits were activated by spinal manipulation compared to sham control.

Motor response time reduced dramatically in the spinal manipulation group both after spinal manipulation (baseline vs. pre-acquisition) and motor acquisition (pre- vs. post-acquisition) without notable improvements to accuracy. In contrast, neck pain control subjects improved accuracy without reduction to response time after sham control (baseline vs. pre-acquisition) and moderate reduction to response time following motor acquisition (pre- vs. post-acquisition). Healthy controls only marginally reduced response time and had no change to accuracy. It is possible that a greater inhibitory drive in neck pain controls may account for their increased accuracy. In contrast, spinal manipulation may have decreased descending inhibitory drive, resulting in faster movements, at the cost of decreased accuracy. However, correlation was not found between CBI and response time or accuracy in any group.

We selected a task that was novel to participants and had limited predictability. Doyon et al. [50] showed that for early stages of motor sequence learning (similar to the task that we had the participants complete), the deep cerebellar nuclei and cortex is more active and in later stages of motor learning the cortico-striatal layers of the brain are more active. We only tested the participants for a brief period of time early in their learning stage when the cerebellum would be more active, as described in the Doyon et al. [45] study.

Previous work [31] showed that the CBI reduced with a combined intervention of spinal manipulation and motor training in recurrent neck pain subjects. Unlike the current study, Daligadu et al. [31] showed that healthy subjects did not reduce CBI following motor training. Likely this was because the task was not hard enough. The current study used a more challenging task shown to reduce CBI in healthy subjects [20]. It was also necessary to include a neck pain control group to act as a control for the manipulation. Without this group, it is impossible to know whether neck pain or its treatment lead to reduced CBI following motor training [31]. In the current study, we found that treatment with spinal manipulation lead to reduced CBI following motor acquisition, whereas neck pain subjects who had sham control did not reduce CBI. In this study, we did not investigate long-term changes to CBI. We also do not know if significant CBF following manipulation and motor acquisition signifies a clinical benefit, but the fact the healthy controls showed less CBI in response to motor learning demonstrates that the CBF observed in the manipulation group is in the direction of the healthy response [20].

In conclusion, subclinical recurrent neck pain participants did not reduce CBI following the completion of a novel motor sequence task compared to healthy participants, and a single session of spinal manipulation restored the capacity for reduced CBI and produced CBF. This study suggests that people with ongoing neck pain may have impaired early processing and/or integration of sensory input from the neck which influences motor processing in the cerebellum and/or the primary motor cortex, which is restored to levels even above that of healthy participants by spinal manipulation. These findings have relevance to the recurrence and potential development of chronic problems and suggest a possible preventative role for spinal manipulation.

## Supporting information

S1 Table CBI data for all participants. CBI, cerebellar inhibition; CBI_50_+5%, cerebellar inhibition with conditioning stimulus intensity 5% greater than the conditioning stimulus intensity for CBI alone; CBI_50_+10%, cerebellar inhibition with conditioning stimulus intensity 10% greater than the conditioning stimulus intensity for CBI alone; MEP, motor evoked potential; pre, pre motor acquisition; post, post motor acquisition; RMT, resting motor threshold.
(XLSX)

S2 Table Motor performance data for all participants. Baseline, baseline motor performance before TMS or motor acquisition; pre, motor performance prior to motor acquisition; post, motor performance following motor acquisition.
(XLSX)
